# Two novel nomograms based on inflammatory cytokines or lymphocyte subsets to differentially diagnose severe or critical and Non-Severe COVID-19

**DOI:** 10.18632/aging.203307

**Published:** 2021-07-19

**Authors:** Zhijun Li, Nan Jiang, Xinwei Li, Bo Yang, Mengdi Jin, Yaoyao Sun, Yang He, Yang Liu, Yueying Wang, Daoyuan Si, Piyong Ma, Jinnan Zhang, Tianji Liu, Qiong Yu

**Affiliations:** 1 Department of Epidemiology and Biostatistics, School of Public Health, Jilin University, Changchun 130021, China; 2 Department of Emergency, China-Japan Union Hospital of Jilin University, Changchun 130021, China; 3 Institute of Organ Transplantation, Tongji Hospital, Tongji Medical College, Huazhong University of Science and Technology, Wuhan 430000, China; 4 Department of Cardiology, China-Japan Union Hospital of Jilin University, Changchun 130021, China; 5 Department of Critical Care Unit, China-Japan Union Hospital of Jilin University, Changchun 130021, China; 6 Department of Neurosurgery, China-Japan Union Hospital of Jilin University, Changchun 130021, China

**Keywords:** COVID-19, SARS-CoV-2, cytokine profiles, lymphocyte subsets, nomogram

## Abstract

We intend to evaluate the differences of the clinical characteristics, cytokine profiles and immunological features in patients with different severity of COVID-19, and to develop novel nomograms based on inflammatory cytokines or lymphocyte subsets for the differential diagnostics for severe or critical and non-severe COVID-19 patients. We retrospectively studied 254 COVID-19 patients, 90 of whom were severe or critical patients and 164 were non-severe patients. Severe or critical patients had significantly higher levels of inflammatory cytokines than non-severe patients as well as lower levels of lymphocyte subsets. Significantly positive correlations between cytokine profiles were observed, while they were all significantly negatively correlated with lymphocyte subsets. Two effective nomograms were developed according to two multivariable logistic regression cox models based on inflammatory cytokine profiles and lymphocyte subsets separately. The areas under the receiver operating characteristics of two nomograms were 0.834 (95% CI: 0.779–0.888) and 0.841 (95% CI: 0.756–0.925). The bootstrapped-concordance indexes of two nomograms were 0.834 and 0.841 in training set, and 0.860 and 0.852 in validation set. Calibration curves and decision curve analyses demonstrated that the nomograms were well calibrated and had significantly more clinical net benefits. Our novel nomograms can accurately predict disease severity of COVID-19, which may facilitate the identification of severe or critical patients and assist physicians in making optimized treatment suggestions.

## INTRODUCTION

Severe acute respiratory syndrome coronavirus 2 (SARS-CoV-2), a member of β-Coronavirus (CoV) lineage B causing the outbreak of coronavirus disease 2019 (COVID-19), was first identified and reported in Wuhan, China in December, 2019 [1, 2]. SARS-CoV-2 has affected the world to varying degrees to become a major cause of significant morbidity and mortality globally, and the outbreak of COVID-19 has been declared as a global pandemic by WHO on March 11, 2020 [3]. As of Feb 25^th^, 2021, the total number of COVID-19 patients globally reported to WHO had reached 112,209,815 of confirmed cases and 2,490,776 deaths in 223 countries, areas or territories. The clinical and epidemiological features of patients with COVID-19 demonstrate that most first-episode patients are asymptomatic or experience mild to severe respiratory illness [4], and older men with comorbidities were more susceptible to infection and associated with higher disease severity and mortality that might due to their weaker immune functions [5, 6]. Besides, SARS-CoV-2 infection can cause multi-organ failure and systemic manifestations especially in critical illness [7].

The laboratory studies have shown that data of both lymphocyte subsets and cytokine profiles are crucial for diagnosis and treatment of patients with COVID-19. The majority of patients are reported to have lymphopenia, while the most available biomarkers of routine clinical tests including C-reactive protein (CRP), neutrophils and lymphocytes ratio (NLR), lactate dehydrogenase and D-dimer were associated with disease severity and could contribute in predicting clinical outcomes in COVID-19 patients [8, 9]. Consistently with SARS and MERS-CoV infections [10, 11], several studies have shown that pro-inflammatory cytokine profiles elevated in patients with COVID-19, and that cytokine storm are directly correlated with pulmonary inflammation, tissue injury and poor prognosis of severe cases [4, 12]. Higher levels of cytokine storm could aggravate the progression of illness in severe COVID-19 patients [7, 13]. Besides, previous studies demonstrated that interleukin-2R (IL-2R)/lymphocyte, interleukin-6 (IL-6) and interleukin-10 (IL-10) might be the potential biomarkers for early diagnosis and indicators for higher risk of disease deterioration when predicting the disease progression of COVID-19 [3, 4, 14]. As for lymphocyte subsets, circulating levels of lymphocyte subsets were suggested to be correlated with clinical features and treatment efficacy of SARS-CoV-2 infection which mainly involve T lymphocytes (particularly CD4^+^ and CD8^+^ T cells), and CD8^+^ T cells alone might be an independent biomarker for the disease severity and treatment efficacy [5, 12, 15]. Therefore, lymphocyte subsets and cytokine profiles seem to be promising for evaluating the disease severity of COVID-19 patients and providing reference for clinical intervention. However, evidence so far has shown inconsistency. A longitudinal analysis of lymphocyte subsets and cytokine profiles in 40 confirmed COVID-19 patients has showed that levels of cytokine profiles and counts of T cells in survivors of severe COVID-19 would return to normal levels comparable with mild patients at a later stage [16]. However, another study which included 25 confirmed COVID-19 patients who were admitted to ICU showed that circulating levels of IL-2, interleukin-4 (IL-4), tumor necrosis factor-α (TNF-α), interferon (IFN-γ) and CRP were not directly correlated with symptom severity of COVID-19 [17]. Thus, the pathophysiology in SARS-CoV-2 infection, especially the correlation of cytokine profiles and immunological features with different disease severity in patients with COVID-19, has not yet been completely understood, and available biomarkers for predicting the disease progression of COVID-19 patients are still limited.

In this study, we performed a comprehensive evaluation of features of 254 COVID-19 patients for the differences in laboratory biomarkers between severe or critical and non-severe patients, including inflammatory markers, cytokine profiles and immunological features. Besides, we developed two novel nomograms for the prediction of disease severity using inflammatory cytokine profiles or lymphocyte subsets data separately. These findings may help us extend our understanding of potential biomarkers associated with disease severity for early identification and guidance of treatment management.

## RESULTS

### Demographic and baseline clinical characteristics of patients with disease severity in COVID-19

A total of 254 patients with confirmed SARS-Cov-2 infection were included in the study and classified into two groups by disease severity. 90 (35.4%) of them were severe or critical patients, and 164 (64.6%) were non-severe patients. Demographic and clinical characteristics of severe or critical and non-severe patients are shown in Table 1. The median age of severe or critical patients (70 years, IQR (64–77)) was significantly higher than non-severe patients (65 years, IQR (55–72)) (*P* < 0.001). Males in severe or critical patients were markedly more common than females (*P* < 0.05). 58.7% of severe or critical patients had at least one comorbidity, with hypertension and diabetes being the most common ones. Median hospitalization time duration for severe or critical patients was 17.5 days (IQR 6.8–29.2) and 23 days (IQR 15–28.8) for non-severe patients (Table 1).

**Table 1:** Differences of clinical characteristics and laboratory findings of patients with COVID-19 on admission.

**Clinical Characteristics**	**Normal Range**	**Median (IQR) or *n*/*N* (%)**		***P* value**
		**Non-severe (164)**	**Severe or Critical (90)**	
**Age, y**		65 (55–72)	70 (64–77)	<0.001
**Sex, *n* (%)**				0.006
Female		91 (55.5%)	33 (36.7%)	
Male		73 (44.5%)	57 (63.3%)	
**Comorbidities, *n*/*N* (%)**				
Any comorbidities		21/38 (55.3%)	37/63 (58.7%)	0.836
Hypertension		15/38 (39.5%)	25/63 (39.7%)	0.983
Diabetes		4/38 (10.5%)	16/63 (25.4%)	0.078
**Symptoms, *n*/*N* (%)**				
Fever		33/38 (86.8%)	53/63 (84.1%)	0.780
Cough		33/38 (86.8%)	53/63 (84.1%)	0.780
Expectoration		26/38 (68.4%)	39/63 (61.9%)	0.529
Dyspnea		25/38 (65.8%)	41/63 (66.1%)	0.972
Myalgia or fatigue		28/38 (73.7%)	36/63 (57.1%)	0.135
Chest tightness		19/39 (48.7%)	36/63 (57.1%)	0.422
Diarrhea		18/38 (47.4%)	28/63 (44.4%)	0.838
Nausea or vomiting		17/38 (44.7%)	13/63 (20.6%)	0.014
Headache		21/38 (55.3%)	21/63 (33.3%)	0.038
Abdominal pain		17/38 (44.7%)	11/63 (17.5%)	0.005
Muscle soreness		18/38 (47.4%)	23/63 (36.5%)	0.303
Sore throat		13/38 (34.2%)	17/63 (27.0%)	0.503
Hemoptysis		12/38 (30.8%)	16/63 (25.4%)	0.649
**Hospitalization period, d**		23 (15–28.8)	17.5 (6.8–29.2)	0.013
**Blood routine**				
Leucocytes, × 10^9^/L	3.5–9.5	5.66 (4.51–6.93)	9.26 (6.06–12.75)	<0.001
Increased		12 (7.3%)	44 (48.9%)	<0.001
Decreased		9 (5.5%)	5 (5.6%)	
Neutrophils, × 10^9^/L	1.8–6.3	3.84 (2.64–4.86)	7.94 (4.83–11.70)	<0.001
Increased		20 (12.2%)	55 (61.1%)	<0.001
Decreased		8 (4.9%)	2 (2.2%)	
Lymphocytes, × 10^9^/L,	1.1–3.2	1.16 (0.85–1.55)	0.64 (0.44–0.87)	<0.001
Decreased		75 (45.7%)	76 (84.4%)	<0.001
NLR		3.2 (2.0–5.2)	11.5 (6.7–20.7)	<0.001
Decreased		23 (14.0%)	1 (1.1%)	<0.001
Increased		34 (20.7%)	72 (80.0%)	
Monocyte, × 10^9^/L	0.1–0.6	0.51 (0.41–0.63)	0.44 (0.28-0.64)	0.024
Platelets, mean (SD), × 10^9^/L	125–350	252 ± 95	181 ± 96	<0.001
Increased		27 (16.5%)	4 (4.4%)	<0.001
Decreased		11 (6.7%)	27 (30.0%)	
**Infectious biomarkers**				
PCT, ng/mL	0.02–0.05	0.04 (0.03–0.08)	0.16 (0.09–0.56)	<0.001
ESR, mm/h	0–20 mm/H	41.0 (20.0–64.0)	36 (17.0–64.2)	0.610
cTnI, pg/mL	≤15.6	4.0 (2.0–9.5)	43.6 (6.6–305.6)	<0.001
NT-proBNP, pg/mL	<738	174 (66–377)	746 (229.5–2302)	<0.001
CRP, mg/L	<1	10.6 (1.9–40.3)	86.3 (43.6–174)	<0.001
Increased		130 (79.3%)	86 (95.6%)	0.007
**Organ Function**				
ALT, U/mL	≤ 33	23 (14–40)	29.5 (18.0–44.2)	0.011
AST, U/mL	≤ 32	24 (18–33.8)	42 (23.8–62.8)	<0.001
TP, g/L	64–83	69.9 (65.9–73.6)	65.3 (59.5–70.4)	<0.001
Albumin, g/L	35–53	35.4 (32.4–38.7)	30.8 (27.7–33.1)	<0.001
Globulin, U/mL	25–35	33.7 (30.5–37.8)	34.8 (30.6–39.2)	0.411
Glu, mmol/L	4.11–6.05	5.72 (5.13–6.94)	7.37 (5.9–10.4)	<0.001
TBIL, umol/L	≤ 2	9.2 (7.0–13.2)	13.4 (9.2–18.4)	<0.001
DBIL, umol/L	≤ 8	3.9 (3.0–5.3)	6.2 (4.1–9.9)	<0.001
IBIL, umol/L	≤ 12.9	5.1 (3.9–7.8)	6.2 (4.5–9.1)	0.037
ALP, U/L	35–105	66.5 (54–86)	79.5 (58–105)	0.002
GGT, U/L	6–42	27.5 (18–46.8)	36.5 (20.8–76.2)	0.013
TC, mmol/L	<5.18	3.8 (3.3–4.5)	3.4 (2.9–4.3)	0.006
LDH, U/L	135–214	258 (214–329.8)	480 (325–652)	<0.001
Mb, ng/mL	≤ 106	43.6 (29.8–81.8)	117.1 (60.5–290.2)	<0.001
CK-MB, ng/mL	≤ 3.4	0.7 (0.4–1.2)	1.8 (0.9–5.8)	<0.001
Creatinine, umol/L	45–85	70 (59–84)	85 (67–105.5)	<0.001
**Coagulation function**				
PT, s	11.5–14.5	13.7 (13.2–14.2)	15.3 (14.2–16.7)	<0.001
INR	0.8–1.2	1.04 (0.99–1.09)	1.20 (1.10–1.34)	<0.001
Fibrinogen, g/L	2–4	5.05 (3.65–6.02)	4.56 (3.10–6.13)	0.175
APTT, s	29–42	38.6 (36.3–42.3)	40.1 (35.6–46.2)	0.209
D-dimer, U/mL FEU	<0.5	1.05 (0.40–1.91)	4.60 (1.44–21.00)	<0.001
**Urine routine**				
Urine protein, *n*/*N* (%)	–	61/149 (40.9%)	62/76 (81.6%)	<0.001
USG, *n*, median (IQR)	1.01–1.025	149, 1.015 (1.011–1.019)	76, 1.02 (1.016–1.024)	<0.001
pH, *n*, median (IQR)	4.5–8.0	149, 6.5 (6.0–7.0)	76, 6.0 (5.5–6.5)	0.002
KET, *n*/*N* (%)	–	11/149 (7.4%)	26/76 (34.2%)	<0.001
URO, *n*/*N* (%)	–	2/149 (1.3%)	10/76 (13.2%)	<0.001
**Arterial Blood Gas**				
pH, *n*, median (IQR)	7.35–7.45	40, 7.41 (7.39–7.46)	37, 7.42 (7.38–7.46)	0.984
PaCO2, *n*, mmHg	35–45	39, 40.9 (38.3–43.8)	37, 36.1 (29.9–41.0)	<0.001
PaO2, *n*, mmHg	80–100	39, 142 (90.8–187)	37, 84 (57.8–148.0)	0.004
SB, *n*, mmol/L	21–25	38, 25.8 (24.8–27.9)	37, 23.8 (21.2–25.4)	<0.001
BEecf, *n*, mmol/L	–3–3	39, 2.1 (0.3–3.9)	37, –0.9 (–4.2–1.3)	<0.001
TCO2, *n* mmol/L	24–32	38, 23.2 (21.7–24.6)	37, 20.9 (16.5–23.5)	<0.001
SaO2, *n*, median (IQR)	91.9–99 %	39, 99.2 (97.2–99.6)	37, 96.2 (86.8–99.5)	0.011

Abbreviations: NLR: Neutrophil-to-lymphocyte ratio; PCT: Procalcitonin; ESR: Erythrocyte sedimentation rate; cTnI: Cardiac troponin I; NT-proBNP: N-terminal of the prohormone brain natriuretic peptide; CRP: C-reactive protein; ALT: Alanine aminotransferase; AST: Aspartate aminotransferase; TP: Total protein; Glu: Glucose; TBIL: Total bilirubin; DBIL: Direct bilirubin; IBIL: Indirect bilirubin; ALP: Alkaline phosphatase; GGT: γ-glutamyl transpeptidase; TC: Total cholesterol; LDH: Lactate dehydrogenase; Mb: Myoglobin; CK-MB: Creatine kinase-MB; PT: Prothrombin time; INR: International normalized ratio; APTT: Activated partial thromboplastin time; USG: Urine specific gravity; KET: Urine ketone body; URO: Urobilinogen; PaCO2: Partial pressure of carbon dioxide; PaO2: Arterial partial pressure of oxygen; SB: Standard bicarbonate; BEecf: Base excess of extracellular fluid; TCO2: Total carbon dioxide; SaO2: Oxygen saturation.

### Differences of laboratory findings between severe or critical and non-severe COVID-19

Table 1 showed general data of indicators as the patients were divided into severe or critical group and non-severe group, which included indicators from blood and urine routine examinations and arterial blood gas analysis, and biomarkers related to organ function, coagulation function and infection. Severe or critical patients presented significantly higher leukocytes and neutrophils counts, elevated neutrophil-to-lymphocyte ratio (NLR) than non-severe patients (all *P* < 0.001), as well as decreased counts of lymphocytes, monocytes and platelets (*P* < 0.05). Compared with non-severe patients, the levels of several infection-related biomarkers were significantly higher in severe or critical patients, including procalcitonin, cardiac troponin I (cTnI), N-terminal prohormone of brain natriuretic peptide (NT-proBNP), C-reactive protein (CRP) (all *P* < 0.001). Also, severe or critical patients had markedly higher levels of serum biochemical indexes including alanine aminotransferase (ALT), aspartate aminotransferase (AST), glucose (Glu), total bilirubin (TBIL), alkaline phosphatase (ALP), γ-glutamyl transpeptidase (GGT), lactate dehydrogenase (LDH), myoglobin, creatine kinase-MB (CK-MB), and creatinine. And they had significantly lower levels of total protein (TP), albumin, total cholesterol (TC) (all *P* < 0.05). Levels of some coagulation factors were significantly elevated in severe or critical cases compared with non-severe patients, including prothrombin time (PT) and D-dimer (all *P* < 0.001).

Compared with non-severe cases, the positive rates of urine protein, urine ketone body (KET) and urobilinogen (URO) in severe or critical cases were significantly higher (all *P* < 0.001). Severe or critical patients almost had significantly lower levels of all indicators in blood gas analysis, including partial pressure of carbon dioxide (PaCO2), arterial partial pressure of oxygen (PaO2), standard bicarbonate (SB), base excess of extracellular fluid (BE), total carbon dioxide (TCO2) and oxygen saturation (SaO2) (all *P* < 0.05) (Table 1).

### Differences of immunologic features in disease severity of COVID-19

**Figure 1:**
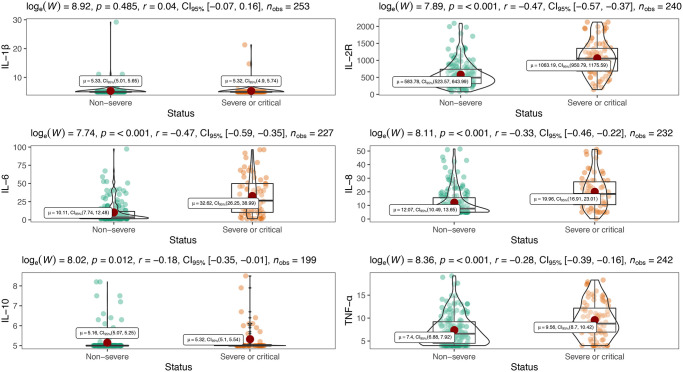
Distribution of peripheral inflammatory cytokines in severe and non-severe patients.

**Figure 2:**
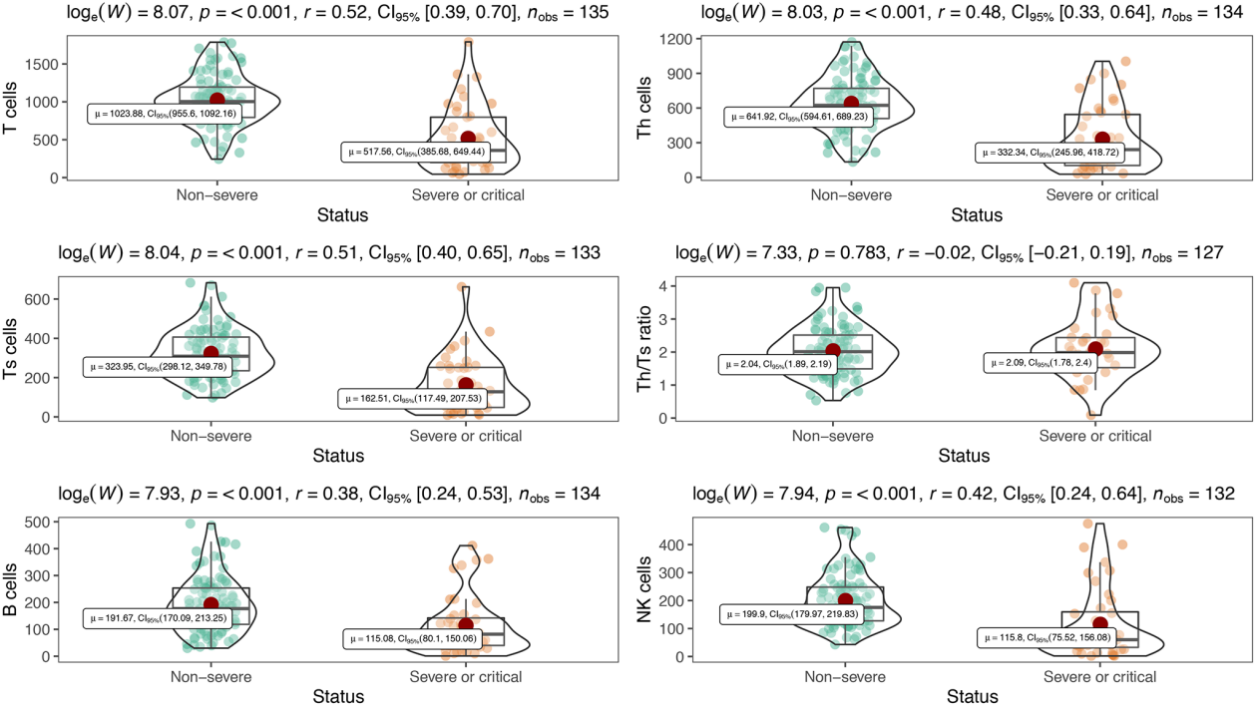
Peripheral lymphocyte subsets between severe and non-severe.

Table 2 showed inflammatory cytokine profiles and lymphocyte subsets in severe or critical patients and non-severe patients with COVID-19. We included interleukin-1β (IL-1β), interleukin-2R (IL-2R), interleukin-6 (IL-6), interleukin-8 (IL-8), interleukin-10 (IL-10) and tumor necrosis factor α (TNF-α) in cytokine profiles. Except for IL-1β which was undetectable (< 5 pg/mL), levels of all other cytokines were significantly increased in severe or critical patients compared with their normal ranges, and markedly higher than those in non-severe patients (all *P* < 0.001) (Table 2, Figure 1). Meanwhile, all lymphocyte subset counts including T, B, Th, Ts and NK cells were significantly decreased to be markedly lower in severe or critical cases (all *P* < 0.001) (Table 2, Figure 2). Nevertheless, there is no statistical significance in Th/Ts ratio between severe or critical and non-severe patients (*P* > 0.05).

**Table 2:** Differences of immune response in patients with COVID-19 on admission.

**Biomarker**	**Normal Range**	***N*, Median (IQR)**		***P* value**
		**Non-severe (164)**	**Severe or Critical (90)**	
**Inflammatory cytokines**				
IL-1β, *n*, pg/mL	<5	164, 5 (5–5)	90, 5 (5–5)	0.742
IL-2R, *n*, U/mL	223–710	164, 498 (318.5–750.8)	90, 1111.5 (728.2–1656.0)	<0.001
Decreased, *n* (%)		17 (10.4%)	2 (2.2%)	<0.001
Increased, *n* (%)		101 (61.6%)	19 (21.1%)	
IL-6, *n*, pg/mL	<7	164, 4.0 (1.5–13.0)	90, 42.2 (12.9–96.3)	<0.001
Increased, *n* (%)		59 (36.0%)	77 (85.6%)	<0.001
IL-8, *n*, pg/mL	<62	164, 7.8 (5.0–16.3)	90, 22.9 (11.8–49.2)	<0.001
Increased, *n* (%)		2 (1.2%)	18 (20.0%)	<0.001
IL-10, *n*, pg/mL	<9.1	164, 5.0 (5.0–5.0)	90, 6.6 (5–13.4)	<0.001
Increased, *n* (%)		10 (6.1%)	39 (43.3%)	<0.001
TNF-α, *n*, pg/mL	<8.1	164, 6.6 (4.5–9.4)	90, 9.8 (7.1–14.1)	<0.001
Increased, *n* (%)		44 (26.8%)	49 (54.4%)	<0.001
**Lymphocyte Subsets**				
T cells + B cells + NK cells, *n*/μL	– –	96, 1435 (1145.5–1718.8)	41, 736 (332–1160.5)	<0.001
T cells (CD3^+^ CD19^–^), *n*/μL	955–2860	96, 1005.5 (794–1231)	41, 359 (198.5–804.5)	<0.001
Decreased, *n*/*N* (%)		37/96 (38.5%)	34/41 (82.9%)	<0.001
B cells (CD3^–^ CD19^+^), *n*/μL	90–560	96, 177 (118.5–256)	41, 84 (40–154)	<0.001
Decreased, *n*/*N* (%)		17/96 (17.7%)	22/41 (53.7%)	<0.001
Th cells (CD3^+^ CD4^+^), *n*/μL	550–1440	96, 632 (509–809.5)	41, 240 (102.5-552.5)	<0.001
Decreased, *n*/*N* (%)		34/96 (35.4%)	31/41 (75.6%)	<0.001
Ts cells (CD3^+^ CD8^+^), *n*/μL	320–1250	96, 314 (237–418)	41, 128 (47–255.5)	<0.001
Decreased, *n*/*N* (%)		49/96 (51.0%)	36/41 (87.8%)	<0.001
NK cells (CD3^–^/CD16^+^ CD56^+^), *n*/μL	150–1100	96, 190 (128–257)	41, 60 (30.5–186.5)	<0.001
Decreased, *n*/*N* (%)		36/96 (37.5%)	28/41 (68.3%)	<0.001
Th/Ts ratio	0.71–2.78	96, 2.04 (1.49–2.59)	41, 2.29 (1.58–3.55)	0.130
Increased, *n*/*N* (%)		16/96 (16.7%)	13/41 (31.7%)	0.137

Abbreviations: IL: Interleukin; TNF: Tumor necrosis factor; Th: helper T cells; Ts: suppressor T cells.

### Correlation analysis between immune-related biomarkers and laboratory-tested indexes

To evaluate the immune responses in patients with COVID-19, especially their impact in organ injury, Spearman rank correlation analyses were performed between immune-related biomarkers with major biochemical indices among patients. We found that there were significantly positive correlations between cytokine profiles. The levels of these cytokine profiles also presented positive correlations with CRP and NLR as well as serum biochemical indexes including Glu, CK, CK-MB, AST and LDH. Besides, levels of serum biochemical indexes were found to be positively correlated with CRP and NLR. On the other hand, the counts of lymphocytes, such as T, B, Th, Ts and NK cells, were significantly negatively correlated with all other indicators including cytokine profiles, serum biochemical indexes and levels of CRP, NLR. ALT was positively correlated with IL-2R, IL-6, NLR and CRP, but it presented no correlation with IL-8, TNF-α and lymphocyte subsets. No correlations were observed between Glu and AST with Th/Ts ratio. Additionally, we found positive correlations between TC with lymphocyte subsets, while negative correlation with all cytokine profiles, CRP, NLR and Th/Ts ratio (Supplementary Figure 1).

### Evaluation of diagnostic value in immune-related biomarkers for predicting disease severity of COVID-19

**Figure 3:**
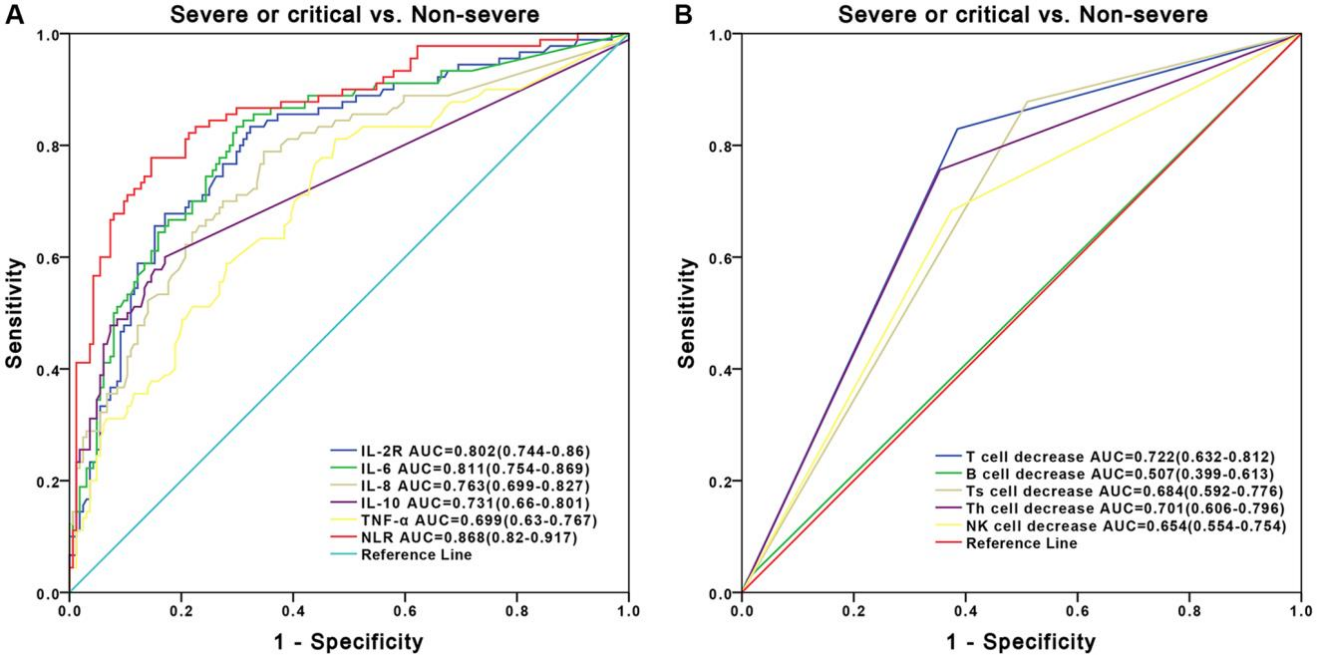
**ROC curve of inflammatory cytokine profiles and lymphocyte subsets for prediction of severe or critical COVID-19.** (**A**) Performance of ROC curves of inflammatory cytokine profiles in predicting the severity of COVID-19. (**B**) Performance of ROC curves of lymphocyte subsets in predicting the severity of COVID-19.

Receiver operating characteristic (ROC) analyses was performed for evaluating the diagnostic value of cytokines and lymphocyte subsets in predicting disease severity of COVID-19. The areas under ROC curves (AUROCs) were 0.868 for NLR, 0.811 for IL-6, 0.802 for IL-2R, 0.763 for IL-8, 0.731 for IL-10 and 0.699 for TNF-α when performing prediction of severe or critical COVID-19 cases (Figure 3A). Besides, the AUROCs were 0.722 for T cells decrease, 0.701 for Th cells decrease, 0.684 for Ts cells decrease, 0.507 for B cells decrease and 0.654 for NK cells decrease (Figure 3B).

### Nomograms development and validation for predicting the disease severity of COVID-19

**Figure 4:**
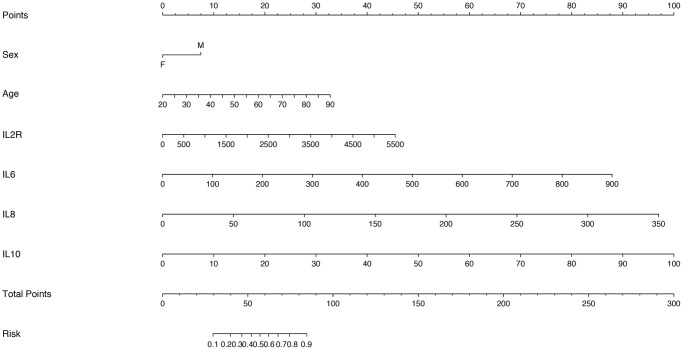
Nomogram constructed by using inflammatory cytokines for prediction of disease severity of COVID-19.

**Figure 5:**
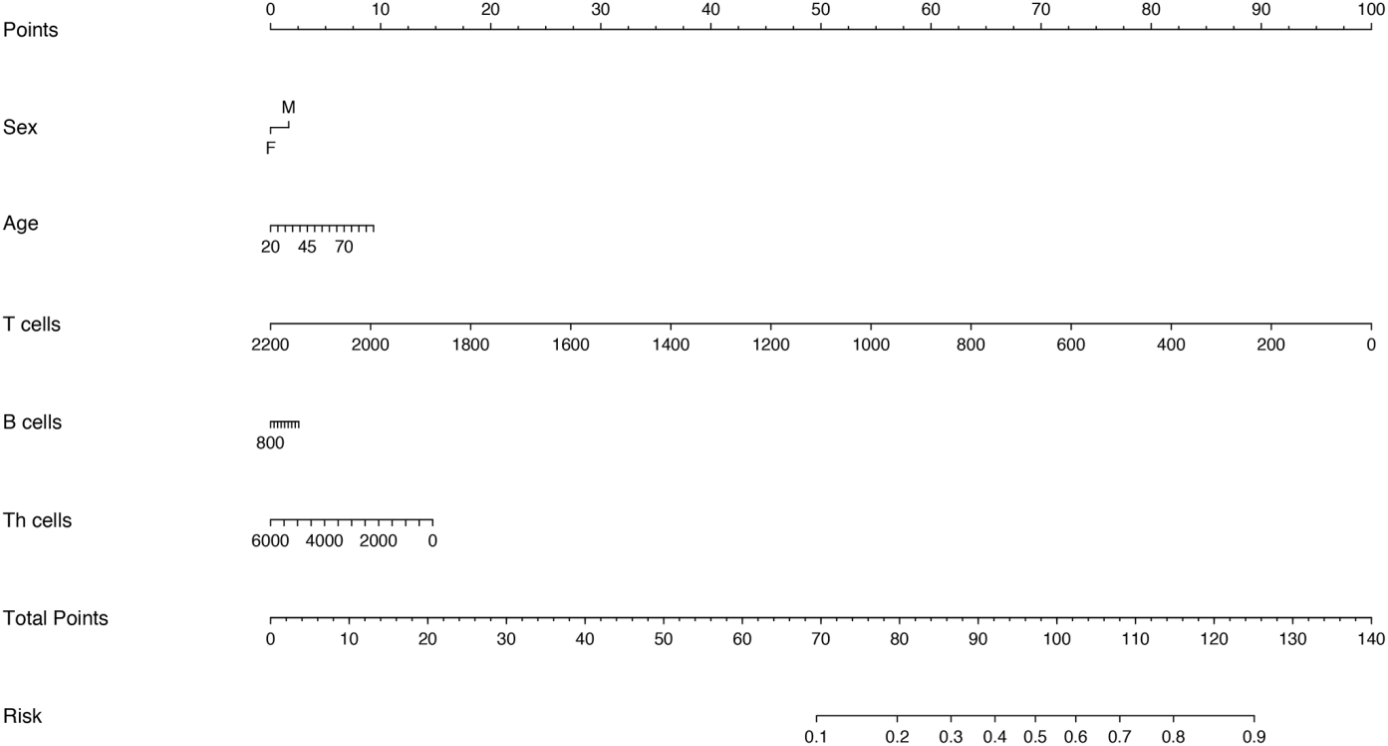
Nomogram constructed by using lymphocyte subsets for prediction of disease severity of COVID-19.

Considering that there was the strong multicollinearity between inflammatory cytokine profiles and lymphocyte subsets, we established two logistic regression models based on inflammatory cytokine profiles and lymphocyte subsets separately. Multivariable analysis models showed that age, IL-2R, IL-6, IL-8, IL-10 and T cells could be the independent risk factors for disease severity (Supplementary Tables 1 and 2). Therefore, we also established two nomograms for predicting the disease severity of COVID-19 based upon the factors screened by the multivariable analyses (Figures 4 and 5), which were used to estimate the probability of higher disease severity by projecting the total score to the lower risk scale.

**Figure 6:**
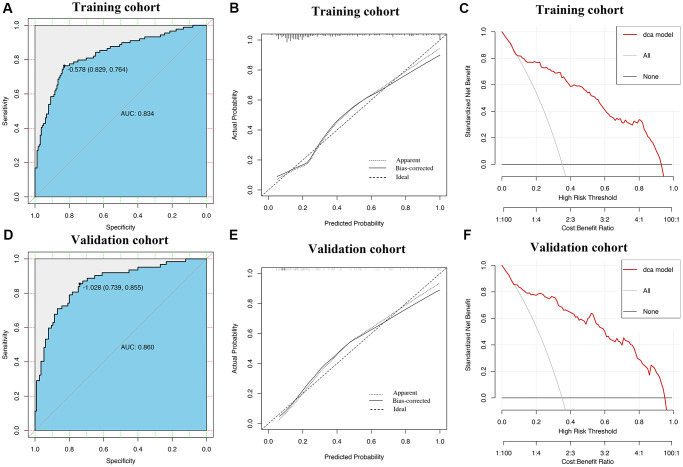
**Construction and validation of the nomogram by using inflammatory cytokines.** The area under receiver operating characteristic curve values, the calibration curve and decision curve analysis in training set (**A**–**C**) and in validation set (**D**–**F**) are shown.

**Figure 7:**
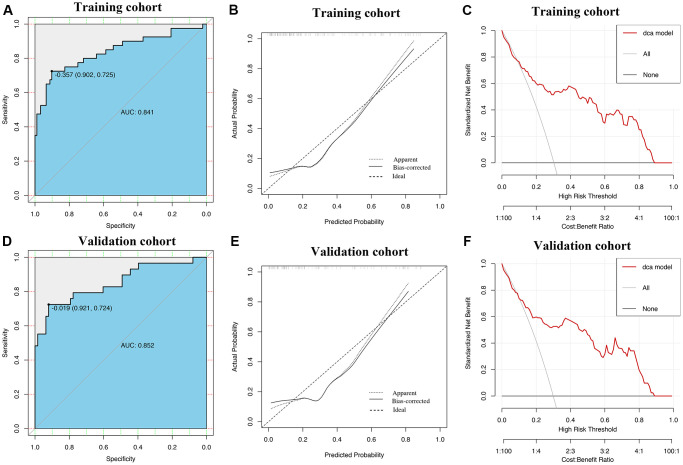
**Construction and validation of the nomogram by using lymphocyte subsets.** The area under receiver operating characteristic curve values, the calibration curve and decision curve analysis in training set (**A**–**C**) and in validation set (**D**–**F**) are shown.

ROC curves showed that AUROCs for the two constructed diagnostic nomograms were 0.834 (95% CI: 0.779–0.888) and 0.841 (95% CI: 0.756–0.925) (Figure 6A, Figure 7A), and the nomograms both exhibited robust discrimination ability. The bootstrapped-concordance indexes of two nomograms were 0.834 and 0.841. The calibration curves of two nomograms (Figure 6B and Figure 7B) demonstrated that they could lead to comparable prediction results for the probability of disease severity that were also quite similar to the actual probability. Besides, the two models were well calibrated with Hosmer-Lemeshow goodness-of-fit results of 5.49 (*p* = 0.703) and 14.69 (*p* = 0.065). Decision curve analyses (DCA) results showed that the two nomograms provide more benefits for practical application (Figure 6C and Figure 7C). In the validation set, the bootstrapped-concordance indexes of two nomograms were 0.860 (0.802–0.919) and 0.852 (0.757–0.946). The calibration curves of two nomograms produced similar results between the predicted probability of disease severity and the actual probability, and DCA results validated the clinical usefulness of the nomograms (Figure 6D–6F and Figure 7D–7F).

## DISCUSSION

Both clinical and immunologic features of patients with COVID-19 have been reported recently [18–21], but how we can make use of these data in differential diagnosis for severe or critical COVID-19 patients still remains a challenge. Besides, there is still insufficient knowledge regarding the immunological indicators related to the mechanism and disease severity of COVID-19. Here, our study on COVID-19 patients at severe or critical and non-severe stages have mainly demonstrated the following findings. First, older males are more susceptible to SARS-CoV-2 infection than females, which was consistent with previous studies [6, 22–25]. Second, the levels of inflammatory cytokines were significantly higher in severe or critical patients than those in non-severe patients while the levels of lymphocyte subsets were significantly lower. Finally, two newly-developed diagnostic nomograms based on inflammatory cytokine profiles and lymphocyte subsets were separately constructed with AUROCs of 0.834 and 0.841. Both of them exhibited robust discriminative ability, which were well improved compared to using blood marker alone (Figure 3). The constructed nomograms could potentially be applied in clinic to differentially diagnose severe or critical and non-severe COVID-19 patients.

In terms of laboratory findings, lymphopenia and increased levels of NLR and infection-related biomarkers were the most common features in severe or critical patients compared to non-severe patients, which was similar to the results in SARS and MERS infections and consistent with previous studies as well [5]. Besides, we found levels of ALT, AST, Glu, TBIL, ALP, GGT, LDH, myoglobin, CK-MB, creatinine and coagulation factors were significantly higher in severe or critical patients than in non-severe patients. Significantly lower levels of TP, albumin, TC and blood gas indicators found in severe or critical patients were also consistent with previous studies [14, 18, 26, 27]. The phenomenon can be explained by the finding of previous study that cytokine storm induced by SARS-CoV-2 infection would cause hepatotoxicity and subsequently critical hypoalbuminemia, which are correlated with inflammatory responses and result in high disease severity and mortality of COVID-19 [26]. In terms of urine routine, the positive rates of urine protein, urine ketone body and urobilinogen in severe or critical cases were significantly higher than non-severe patients. Moreover, we observed that CRP and NLR were significantly positive correlated with Glu, CK, CK-MB, AST and LDH. CRP and NLR have been regarded as predictors of systemic inflammation, bacterial or viral infections and tissue injuries [12, 28, 29]. Recently, several studies have demonstrated that they might be useful potential biomarkers for advanced disease progression of COVID-19 while NLR can reflect the degree of imbalance in systemic inflammation and immune response [12, 16, 29]. In longitudinal analysis, NLR and neutrophil-to-CD8^+^ T cell ratio (N8R) have been indicated as important prognostic factors for severe COVID-19 [16]. It was suggested that monitoring laboratory markers and cytokines could be helpful in predicting the progression, improving treatment efficacy and reducing mortality of COVID-19 [3, 21, 29].

We noted that SARS-CoV-2 infection would induce significant rises in peripheral cytokine profiles and reductions in lymphocytes subsets. Levels of cytokine profiles were significantly higher in severe or critical patients who also presented markedly lower of lymphocyte subsets counts compared with non-severe patients, which were consistent with previous studies and similar to the findings in SARS and MERS [5, 15, 30–32]. Specifically, we also analyzed the correlations between these indexes and found that cytokine profiles were all significantly negatively correlated with lymphocytes subsets. Serum biochemical indexes such as Glu, CK, CK-MB, AST, and LDH as well as CRP and NLR were all significantly positively correlated with cytokine profiles but significantly negatively correlated with T, B, Th, Ts and NK cells. ALT was positively correlated with IL-2R, IL-6, NLR and CRP, while it had no correlations with IL-8, TNF-α or lymphocyte subsets. No correlations were observed between Glu and AST with Th/Ts ratio. Additionally, we found TC was positively associated with lymphocyte subsets but negatively associated with cytokine profiles, CRP, NLR and Th/Ts ratio.

It is believed that SARS-CoV-2 infection would induce cytokine storm and dysregulation of immune response, which is similar with SARS infection. SARS-CoV repaid replication and dysregulated type I interferon would cause elevated levels of cytokines and suboptimal T cell responses in SARS-CoV infected mice [33]. Several studies have established theories that IL-2R/lymphocyte, IL-6 and IL-10 can be used as predictors for early identification of disease and prediction of the risk of disease deterioration [3, 4, 34]. These cytokines can be produced at multiple sites of tissue inflammation and released into the circulation by various types of cells during sepsis and acute organ injuries [34, 35]. They may also play important roles in immune response during viral infections as higher levels of cytokine storm is reported to be correlated with more rapid disease progression, resulting in high incidence of immune disorders and mortality [15, 36, 37]. We found that SARS-CoV-2 infection induced evident lymphopenia and lowered the levels of lymphocyte subsets, which affects the maintenance of immune response and associated with disease severity, and these findings were in consistence with results of previous studies [15, 21, 38, 39]. SARS-CoV-2 infection mainly affects T lymphocytes especially CD4^+^ and CD8^+^ T cells, which were significantly decreased and associated with disease severity in COVID-19. CD8^+^ T cell was the lymphocyte subset with the most obvious decline in longitudinal analysis, which might contribute to inhibiting overactive innate immune responses [16, 18, 39]. However, the functions of B and NK cells are often ignored despite the finding that levels of them both decreased in patients. Previous studies suggested that B and NK cells participated in controlling SARS-CoV-2 infection and were associated with disease severity [15].

Besides, based upon the multivariate models using inflammatory cytokines and lymphocyte subsets, we developed two novel nomograms for differentially diagnosis of severe or critical and non-severe COVID-19 patients. Our results showed that the two models improved the diagnostic power compared with the power of models using each blood indicator alone, and AUROCs for the nomograms were greater than those of blood marker alone in the differentially diagnosis of disease severity (Figure 3). Furthermore, the two novel nomograms showed robust discriminative ability. Given the above findings, our study demonstrated that systemic inflammation responses, cytokine storms and dysregulated immune responses occurred and exerted combined effects on the clinical progression in COVID-19, which makes monitoring these biomarkers helpful for predicting the disease severity and reducing mortality of COVID-19.

There were still several limitations in our study. Firstly, a single-center, retrospective study using data from the designated hospital, which mainly receives severe patients in Wuhan, China might cause several potential biases. Secondly, there was insufficient information of possible temporal changes of cytokine profiles and immunological features in SARS-CoV-2 infected patients. Studies of larger sample sizes may better solve the problem. Thirdly, bacterial coinfection might exit in patients with COVID-19 and thus interference the manifestations of their immune responses. Fourth, the nomograms were constructed by retrospective analysis based on data from one single hospital. Despite there were internal validation, we still lacked data for external validation. Though the nomograms demonstrated good performance, they are preferred to be validated by performing a multiple-center prospective study.

## CONCLUSIONS

In summary, blood inflammatory cytokines and lymphocyte subsets may be potential biomarkers for early screening, diagnosis and treatment of severe or critical COVID-19 patients. We proposed two novel nomograms based on inflammatory cytokines and lymphocyte subsets separately that can accurately predict disease severity of COVID-19. We hope that our novel nomograms may facilitate the identification of severe or critical patients and assist physicians in making optimized treatment suggestions.

## MATERIALS AND METHODS

### Study design and participants

The study was approved by the Ethical Committee of Tongji Hospital of Huazhong University of Science and Technology (No.TJ-IRB20200364) and China-Japan Union Hospital of Jilin University (No.2020032607). All 254 patients with COVID-19 were enrolled in the study and admitted to Tongji Hospital of Huazhong University of Science and Technology from Jan 10, 2020 to Mar 11, 2020. Diagnosis, clinical classifications and complication definitions of patients with COVID-19 were made according to China’s Novel Coronavirus Pneumonia Diagnosis and Treatment Guidance (Seventh version) [40]. Patients with SARS-CoV-2 infection were confirmed by qRT-PCR test on swabs or sputum specimens on admission. Patients younger than 18-year or those with infection of HBV, HCV and HIV were excluded in the study.

### Data collection of clinical and laboratory analysis

Demographic characteristics, clinical symptoms and laboratory data of the patients including inflammatory cytokine profiles and lymphocyte subsets analyses on admission were collected and extracted from their electronic medical records. All data were extracted with a unified data collection form and checked by two independent physicians.

### Statistical analysis

SPSS 22.0 and R 3.6.1 software were used for statistical analysis. We presented count data as percentages, and measurement data were described as means (SD) or medians (IQR). Statistical differences were analyzed by Chi-square test or the Fisher exact test for count data, and Mann-Whitney *U*-test or unpaired *t*-test for continuous data. Spearman rank coefficient analyses were performed for determining correlations between the immunologic biomarkers and laboratory-related indexes. The diagnostic values of peripheral inflammatory cytokines and lymphocyte subsets for predicting the disease severity of COVID-19 were evaluated using area under the receiver operating characteristic (AUROC) curves. The potential risk factors of disease severity in COVID-19 patients were examined by multivariable logistic regression model. Before entering the models, the variables were screened by variance inflation factor (VIF) using ‘vif’ function of ‘car’ package, and only variables with VIFs < 5 entered the models. All data were used as training set for the development of the prediction nomograms, and the discrimination power of nomograms were evaluated by the concordance index, ROC curves and calibration plots with 1000 bootstrap resampling. Besides, the calibration of models was assessed by Hosmer-Lemeshow goodness-of-fit test with ‘ResourceSelection’ package. 70% of the original data were randomly selected as the internal validation set for evaluating the application efficacy of the prediction nomograms. *P* < 0.05 indicated statistical significance in all analysis.

## Supplementary Materials

Supplementary Figure

Supplementary Tables
