# Supplementary material for: Two novel nomograms based on inflammatory cytokines or lymphocyte subsets to differentially diagnose severe or critical and Non-Severe COVID-19
Source: Aging (Albany NY). 2021 Jul 19;13(14):17961–77. doi: 10.18632/aging.203307 (PMC8351679; doi:10.18632/aging.203307)
Supplement: Supplementary Figure [file aging-13-203307-s001.pdf]

SUPPLEMENTARY FIGURE

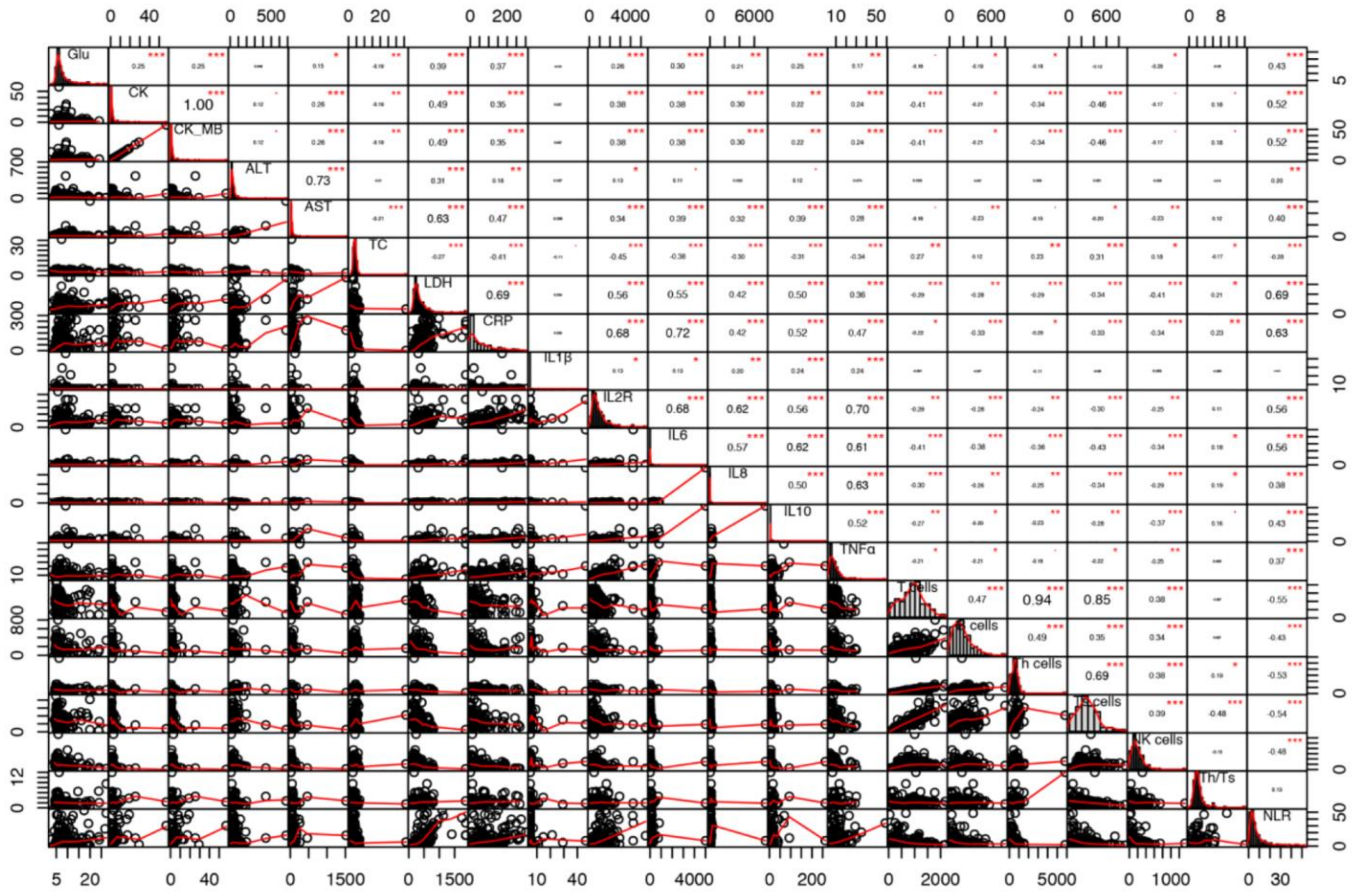

Supplementary Figure 1. Spearman rank correlation analyses between immune-related biomarkers and major organ function indexes among patients of COVID-19.
