# Supplementary material for: Two novel nomograms based on inflammatory cytokines or lymphocyte subsets to differentially diagnose severe or critical and Non-Severe COVID-19
Source: Aging (Albany NY). 2021 Jul 19;13(14):17961–77. doi: 10.18632/aging.203307 (PMC8351679; doi:10.18632/aging.203307)
Supplement: Supplementary Tables [file aging-13-203307-s002.pdf]

SUPPLEMENTARY TABLES

Supplementary Table 1. Multivariate logistic regression analysis of inflammatory cytokines (/mL) in severe or critical COVID-19 patients.

Parameter	β	Wald χ^2	OR (95% CI)	*p*-value
**Intercept**	–5.307	–4.77	–	<0.0001
**Sex (F:M)**	0.5965	1.78	0.551 (0.285–1.063)	0.0753
**Age**	0.0374	2.46	1.688 (1.113–2.559)	0.0138
**IL-2R**	0.0007	2.02	1.588 (1.014–2.486)	0.0434
**IL-6**	0.0078	1.99	1.347 (1.005–1.805)	0.0461
**IL-8**	0.0221	2.44	1.509 (1.085–2.100)	0.0146
**IL-10**	0.0798	2.17	1.118 (1.011–1.237)	0.0300

Abbreviations: M: male; F: female; IL: interleukin.

Supplementary Table 2. Multivariate logistic regression analysis of lymphocyte subsets (/μL) in severe or critical COVID-19 patients.

Parameter	β	Wald χ^2	OR (95% CI)	*p*-value
**Intercept**	1.3696	0.92	–	0.3554
**Sex (M:F)**	0.1306	0.27	1.139 (0.446–2.912)	0.7850
**Age**	0.0106	0.54	1.172 (0.659–2.080)	0.5890
**T cells**	–0.0036	–3.90	0.110 (0.036–0.333)	<0.0001
**B cells**	–0.0003	–0.11	0.959 (0.451–2.037)	0.9130
**Th cells**	–0.0002	–0.24	0.920 (0.471–1.799)	0.8079

Abbreviations: M: male; F: female; Th: helper T cells.
